# Pilot evaluation of a Psychological First Aid online training for COVID-19 frontline workers in American Indian/Alaska Native communities

**DOI:** 10.3389/fpubh.2024.1346682

**Published:** 2024-06-27

**Authors:** Victoria M. O'Keefe, Fiona Grubin, Nainika Vaidya, Tara L. Maudrie, Maisie Conrad, Sophie Neuner, Shardai Jridi, Mary Ann Cook, Kathryn A. Carson, Allison Barlow, Emily E. Haroz

**Affiliations:** ^1^Department of International Health, Social and Behavioral Interventions, Center for Indigenous Health, Johns Hopkins Bloomberg School of Public Health, Baltimore, MD, United States; ^2^Department of Nursing, Red Lake Indian Health Service Hospital, Red Lake, MN, United States; ^3^Department of Epidemiology, Johns Hopkins Bloomberg School of Public Health, Baltimore, MD, United States

**Keywords:** American Indian and Alaska Native, mental health, Psychological First Aid (PFA), Indigenous, COVID-19

## Abstract

**Introduction:**

The COVID-19 pandemic exacerbated mental health concerns and stress among American Indians and Alaska Natives (AI/ANs) in the United States, as well as among frontline workers responding to the pandemic. Psychological First Aid (PFA) is a promising intervention to support mental wellbeing and coping skills during and after traumatic events, such as the COVID-19 pandemic. Since PFA is often implemented rapidly in the wake of a disaster or traumatic event, evidence evaluating its impact is lacking. This paper reports pilot evaluation results from a culturally adapted PFA training designed to support COVID-19 frontline workers and the AI/AN communities they serve during the pandemic.

**Methods:**

This study was designed and implemented in partnership with a collaborative work group of public health experts and frontline workers in AI/AN communities. We conducted a pre-post, online pilot evaluation of a culturally adapted online PFA training with COVID-19 frontline workers serving AI/AN communities. Participants completed a baseline survey and two follow-up surveys 1 week and 3 months after completing the PFA training. Surveys included demographic questions and measures of anxiety, burnout, stress, positive mental health, communal mastery, coping skills, PFA knowledge, confidence in PFA skills, and satisfaction with the PFA training.

**Results:**

Participants included *N* = 56 COVID-19 frontline workers in AI/AN communities, 75% were AI/AN, 87% were female, and most (82%) were between the ages of 30–59. Participants reported high satisfaction with the training and knowledge of PFA skills. Pilot results showed significant increases in positive mental health and social wellbeing and reductions in burnout from baseline to 3 months after completing the PFA training among frontline workers. There were no changes in communal mastery, coping skills, stress, or anxiety symptoms during the study period.

**Discussion:**

To our knowledge, this is the first pilot evaluation of a PFA training designed and culturally adapted with and for AI/AN communities. Given that many AI/AN communities were disproportionately impacted by COVID-19 and prior mental health inequities, addressing acute and chronic stress is of crucial importance. Addressing traumatic stress through culturally adapted interventions, including Indigenous PFA, is crucial to advancing holistic wellbeing for AI/AN communities.

## Introduction

The COVID-19 pandemic illuminated and widened existing health inequities driven by social determinants of health, especially among minoritized communities in the United States (U.S.). Settler colonialism and related systemic racism have propagated harmful policies and practices that undergird American Indian/Alaska Native (AI/AN) health inequities that were exacerbated by the pandemic (1). Reservation communities experienced additional disproportionate impacts of COVID-19 driven by limited health care infrastructure and resources necessary to mitigate risk (e.g., access to clean, running water) (2, 3). Data show that AI/ANs contracted COVID-19 more often than non-Hispanic White populations in the U.S. and experienced some of the highest mortality rates due to COVID-19 (4, 5). The COVID-19 pandemic has also greatly impacted mental, emotional, and spiritual health for AI/AN communities (6). As lockdowns and physical distancing were implemented during the pandemic, traditional healing practices were often disrupted (7). During the pandemic, the emotional wellbeing of many AI/AN peoples living in urban areas worsened, and negative impacts on emotional health were related to concerns about the pandemic's impact on Tribal cultures (e.g., fears related to loss of traditional languages and Elders, decreased participation in cultural practices) (8). There is also evidence that historical trauma contributed to worsening mental health outcomes among AIs during the pandemic by increasing levels of perceived stress (9).

Despite these challenges, AI/AN communities have exhibited and continue to project tremendous resilience during and after the COVID-19 pandemic. Within many AI/AN communities, cultural values inherently shape responses to events like the pandemic, whereby people take collective care of one another (10). For example, AI/AN communities have been national leaders in distributing and administering COVID-19 vaccines and through leveraging cultural values and Tribal sovereignty, AI/AN communities have exhibited the highest vaccine uptake rate of any racial/ethnic group in the U.S. (10).

Frontline workers (e.g., health care workers) were vital to protecting AI/AN communities during the COVID-19 pandemic. The mental health of health care workers is critical to a stable pandemic response (11). Psychological First Aid (PFA) is a tool to provide support to individuals in situations of crisis or trauma with the goal of preventing and reducing additional harm (12, 13). PFA is a promising intervention to support mental wellbeing and coping skills during and after the COVID-19 pandemic. As it is often implemented rapidly and in the wake of a disaster or traumatic event, data evaluating PFA and its impact are limited (14–17). A scoping review of PFA noted that while trainings can increase knowledge, evidence evaluating other outcomes is limited (16). A systematic review exploring PFA efficacy highlighted its impact on reducing anxiety, depression, and post-traumatic stress symptoms and improving mood, safety, and connectedness, but that causal inference was limited by a high risk of bias (17). In addition, there is a dearth of research on the use of PFA with AI/AN communities.

Many AI/AN communities and researchers note the importance of culturally appropriate resources for mental health among AI/AN peoples that incorporate and center cultural values, as AI/AN conceptualizations of health and wellness tend to differ significantly from Western models and mental health treatments (18, 19). To support the wellbeing of COVID-19 frontline workers in AI/AN communities, and in response to experiences shared by colleagues working on the frontlines in their communities, the Johns Hopkins Center for Indigenous Health (CIH) culturally adapted a PFA resource guide and online training (20). The purpose of this paper is to describe the results of a pilot evaluation of this culturally adapted, online PFA training for frontline workers responding to the COVID-19 pandemic in AI/AN communities. Specifically, we aimed to assess satisfaction with the PFA training, its impact on participant knowledge related to PFA, confidence in using PFA skills, and changes among frontline workers' anxiety, coping skills, level of burnout, communal mastery, stress, and positive mental health.

## Methods and materials

### Study design and approach

We employed an observational pre-post pilot evaluation design of the culturally adapted PFA training with AI/AN and other frontline workers responding to the COVID-19 pandemic in AI/AN communities. In line with a community-based participatory approach (CBPR) (21) we engaged a collaborative work group (CWG) in culturally adapting a PFA training (“Basic Psychosocial Skills: A Guide for COVID-19 Responders”) (22) to ensure its relevance and use with COVID-19 frontline workers in AI/AN communities. This CWG was comprised of six AI/AN people and one non-AI/AN person, all of whom possess expertise related to AI/AN public and mental health. Cultural adaptations to the PFA training primarily centered around updating the language to be more culturally appropriate, as well as adding cultural values, teachings, and practices often shared across AI/AN communities. The CWG guided us to an overall theme of “caring for all of our relations,” which reflects cultural values across AI/AN communities. The final “Psychological First Aid for COVID-19 Frontline Workers in American Indian/Alaska Native Communities” includes an online resource guide and training with four modules. The first module, “Your and Your Relatives' Wellbeing,” includes content validating stressors among frontline workers, describing symptoms of stress, and ways to cope with stress and take care of oneself. Module 2, “Supportive Communication in Everyday Interactions,” outlines tips and ideas for strengthening virtual and in-person communication. Module 3, “Offering Practical Support to Community Members,” discusses resources, needs, and ideas for sharing this information with others and helping them solve problems. Module 4, “Supporting Everyone in Our Communities,” describes priority groups (e.g., Elders) that might need additional support during the pandemic and ideas for supporting them. In addition, the training includes three video case examples that demonstrate application of the PFA skills highlighted in the modules. Case examples were co-created with the CWG to ensure they reflected the experiences of frontline workers in AI/AN communities and addressed cultural values of AI/AN communities. Further details of the CWG and PFA cultural adaptation process are described in a previous publication (20).

Following completion of the culturally adapted online guide and training, the CWG reviewed the evaluation study design and assisted the study team with creating a measure of confidence in using PFA skills learned during the training. The study team facilitated this process by asking CWG members “what are all of the positive skills, knowledge, and competencies you would want a participant to gain from taking this PFA training?” Their responses were compiled and used to inform item development for this measure. The input of the CWG was also used to create select items in the training satisfaction measure. In line with core tenets of CBPR with Indigenous communities, the cultural adaptation process of PFA and planning the pilot evaluation focused on strengths, partnering with the CWG at all stages, and co-learning, including inviting CWG members to co-author this manuscript (23).

### Participants

Eligible participants included adults 18 years or older who identified as a frontline worker responding to the COVID-19 pandemic in an AI/AN community. We defined frontline worker as someone whose role in responding to the pandemic routinely placed them in contact with people who may be positive for COVID-19, including, but not limited to, health care workers, community health workers, and contact tracers (20). Participants were excluded if they had an inability to complete study assessments and training (e.g., due to lack of internet access) or inability to provide informed consent.

### Measures

Study assessments included basic demographic information, mental health (i.e., anxiety, burnout, stress, positive mental health), wellbeing (i.e., communal mastery, coping skills), and PFA-related knowledge and skills, including confidence in those skills and satisfaction with the online PFA training. Table 1 provides an overview of these measures and the timepoints at which they were assessed in the study.

**Table 1:** Outcomes and measures included in study assessments.

**Outcome and measure**	**Description**	**Timepoint**		
		**Baseline**	**Follow-up #1**	**Follow-up #2**
Demographics	22 questions ask about basic demographic information, including age, gender, Tribal affiliation(s), and household information.	X		
Anxiety; PROMIS- anxiety short form (24)	Eight questions ask about how often the respondent has experienced feelings of anxiety over the past week. Response options include never, rarely, sometimes, often, and always. Lower scores indicate less anxiety.	X	X	X
Burnout; Single-item burnout measure (25)	One question asks respondents to indicate their level of burnout on a scale from 1 (I enjoy my work. I have no symptoms of burnout) to 5 (I feel completely burned out and often wonder if I can go on. I am at the point where I may need some changes or may need to seek some sort of help). Higher scores indicate greater levels of burnout.	X	X	X
Stress; Perceived Stress Scale-4 (26)	Four questions ask about thoughts and feelings over the past month related to stress and capacity to handle problems. Response options include never, almost never, sometimes, fairly often, and very often. Higher scores indicate greater levels of stress.	X	X	X
Positive mental health; Mental Health Continuum (short form) (27, 28)	14 questions ask about positive feelings respondents have experienced during the past month. Respondents are asked to report the frequency of these feelings ranging from never to every day. Higher scores indicate greater levels of positive mental health.	X	X	X
Communal mastery; Communal mastery scale (29, 30)	10 questions from the Multicultural Mastery Scale (which is originally 15 questions) adapted for another study with input from local Apache researchers ask about communal mastery. Responses range from strongly agree to strongly disagree. Lower scores indicate greater levels of communal mastery.	X	X	X
Coping skills; Brief COPE measure (31)	28 questions ask about strategies for managing stress and respondents report how frequently they use these strategies on a four-point scale. Higher scores on each subscale indicate engaging more in each type of coping strategy.	X	X	X
PFA knowledge; Four short quizzes based on each PFA training module	19 multiple choice and true/false questions ask about information shared in the PFA training to assess respondents' knowledge of each of the four training modules: Module 1: Your and your relatives' wellbeing Module 2: Supportive communication in everyday interactions Module 3: Offering practical support to community members Module 4: Supporting everyone in our communities. Higher scores represent greater competency with PFA knowledge.	X	X	X
Confidence in PFA skills	10 questions ask about confidence in specific PFA related skills that the training is intended to teach. Responses range from 1 (strongly disagree) to 5 (strongly agree). This measure was developed collaboratively with the CWG. Higher scores indicate more confidence in skills.	X	X	X
Satisfaction with PFA training (32)	15 questions adapted from a measure of program satisfaction ask respondents to indicate their level of satisfaction with the PFA training content and delivery after completing the training. Higher scores indicate greater levels of satisfaction.		X	X

### Procedure

Participants were recruited by sharing print and virtual copies of a recruitment flier. Virtual copies were emailed to relevant listservs by study team members and print copies were shared with CIH office locations to post around the office and community as appropriate. The research team asked CWG members to share recruitment information with their networks and made announcements about the study at larger CIH virtual meetings attended by partnering communities and frontline workers. Broader recruitment strategies involved advertising the study through CIH social media posts on Facebook, Instagram, and Twitter. Recruitment materials directed interested participants to an email account managed by a research coordinator and two graduate student research assistants. Potential participants who emailed study staff were provided with a description of study participation and a link to complete an eligibility screening. If deemed eligible, participants were emailed a link to complete an online consent form and baseline survey. Study staff corresponded with potential participants to answer questions and emailed a maximum of three reminders to anyone who expressed interest but never replied or filled out the eligibility survey.

**Figure 1:**
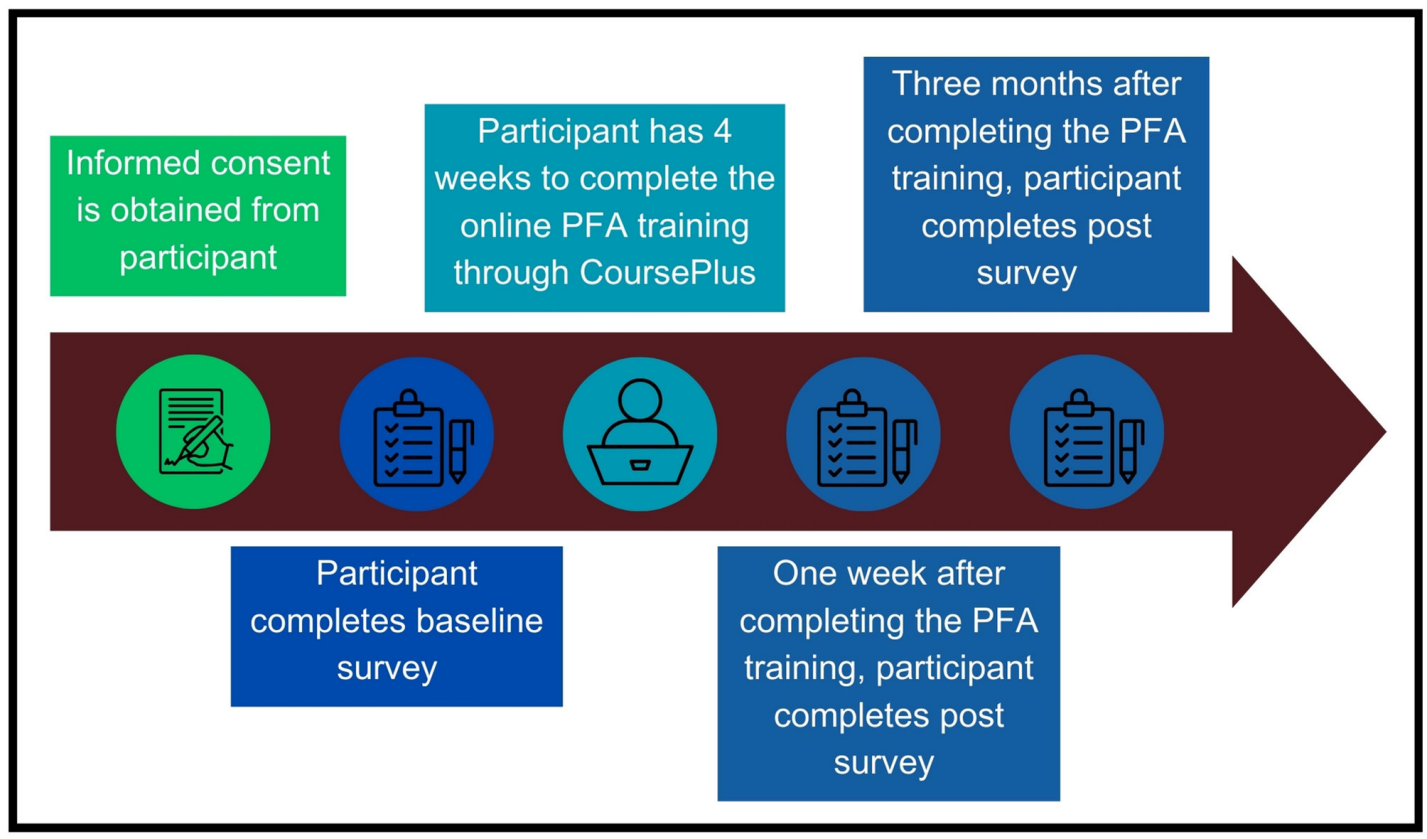
Study implementation.

Following informed consent, participants completed a baseline survey online using REDCap, an electronic data collection platform (33, 34). Upon completion of the baseline survey, REDCap was programmed to email participants information about the online training, which was hosted on CoursePlus, a virtual learning platform for the Johns Hopkins Bloomberg School of Public Health. Participants were notified they had 4 weeks to complete the training. The research coordinator and student research assistants tracked training completion progress regularly and sent up to three reminders to each participant during the 4-week period to complete the training. Participants who completed the training were emailed two follow-up surveys to complete in REDCap, scheduled for 1 week post-training completion and 3 months post-training completion, respectively. Study staff emailed participants reminders related to these surveys ahead of their scheduled dates to discourage attrition and followed-up after scheduled survey due dates had passed with email reminders as necessary to encourage completion. If preferred, participants had the option to complete study assessments via phone with a study team member, though no participants requested this option. Participants were mailed a $15 gift card for completing the baseline survey and each of the two follow-up surveys. Participants who completed all three surveys and the online training (i.e., the entire study) received an extra $30 gift card to encourage completion of the entire study, for a total of up to $75 in compensation. Figure 1 illustrates the procedural flow of the study from a participant's point of view.

### Ethical considerations

This study was reviewed and approved by the Johns Hopkins Bloomberg School of Public Health Institutional Review Board (protocol #15690) and by the Navajo Nation Human Research Review Board (protocol NNR-21.405). Given the rapid timeline for implementing this study during the COVID-19 pandemic, we received approval to enroll participants from Navajo Nation in three of five Agencies within Navajo Nation. The study's eligibility screening included questions to screen out potential participants from non-approved agencies within Navajo Nation. Study staff masked participant identities from PIs to reduce any potential conflict of interest for potentially eligible participants employed by CIH.

### Data analysis

Data were exported from REDCap to MS Excel for review and then imported into SAS version 9.4 (SAS Institute, Inc., Cary, NC) for analysis. Frequency distributions and ranges for individual variables were generated to identify potential errors and outliers in the data, and to identify variables for recoding into larger groups, such as age group and level of education. Cronbach's alpha was calculated for each mental health outcome instrument (i.e., burnout, communal mastery, coping skills, anxiety, positive mental health, perceived stress) and ranged from 0.66 to 0.94. Across the measures of training outcomes, Cronbach's alpha ranged from 0.65 to 0.87. Summary scores for each instrument were calculated and the distributions were assessed for normality. Many of the measures were not normally distributed and the sample size was not large enough to disregard non-normality. Therefore, non-parametric statistics (medians, interquartile ranges (IQRs), and Wilcoxon signed rank tests) were used to compare the baseline data to one-week and three-month follow-up timepoints. All tests were two-sided and significance was set at *p* < 0.05. No adjustment was made for multiple comparisons. Given the non-parametric approach used and missing data mechanism, we examined the impact of missing data due to loss to follow-up on our outcome models through a sensitivity analysis. We performed this analysis by imputing values corresponding to no change for each individual and their missing follow-up data and then compared inferences between this model that assumes no change and the reported model in the manuscript.

## Results

### Sample demographics

Table 2 summarizes sociodemographic characteristics of *N* = 56 participants in the sample. The majority of participants (87%) were female and between 30 and 59 years old (82%). Most participants were AI/AN and 42 participants (75%) reported at least one Tribal affiliation. The total sample represented 30 different Tribal Nations. In addition, 11 participants (20%) indicated they were not AI/AN, and three participants declined to answer this question. Approximately half of participants (46%) reported their occupation as a type of medical/health care worker (e.g., doctor, nurse), and 25% identified as a community health worker/representative. Fifty-five percent of the sample indicated their workplace was on a Tribal reservation while 22% reported working in an urban or suburban area. Participants indicated a variety of levels of education: 17% had completed some high school, a high school diploma, or some college; 20% had an associate's degree; 34% had a bachelor's degree; 29% held a graduate degree (including medical degrees); and 2% reported holding some type of medical assistant certificate. Reported size of household ranged up to seven total people, with an average of three household members. On average, participants had one child living with them. Approximately half (52%) noted that at least one person living in their household was at high risk for COVID-19. While only 5% of the sample reported having difficulty obtaining enough drinking water for their household due to COVID-19, 89% of the sample said they had difficulty getting enough washing water for their household due to COVID-19. All participants reported having cell service and most (96%) indicated they had internet access at home. Financially, most participants had just enough or more than enough money to make ends meet (86%) but some (14%) noted having not enough or almost enough money to make ends meet. These demographics suggest that this predominantly AI/AN sample of frontline workers were experiencing many of the same sociodemographic challenges as the communities they serve (e.g., water insecurity, living with people at high risk for COVID-19).

**Table 2:** Sociodemographic characteristics of participants.

**Characteristic**	***N* (%) or median (range)**
**Tribal affiliation**	
Identify with at least one Tribe	42 (75)
Not Indigenous	11 (20)
Declined to answer	3 (5)
**Gender**	
Female	49 (87)
Male	6 (11)
Other	1 (2)
**Age category**	
20–29 years	6 (11)
30–39 years	21 (37)
40–49 years	14 (25)
50–59 years	11 (20)
60–69 years	3 (5)
70–79 years	1 (2)
**Marital status**	
Single	20 (36)
Living with partner	7 (13)
Legally married	23 (41)
Divorced	3 (5)
Widowed	3 (5)
Education:	
Some high school	1 (2)
High school diploma	2 (4)
Some college	6 (11)
Associate's degree	11 (20)
Bachelor's degree	19 (34)
Graduate education	11 (20)
Graduate medical education	5 (9)
Other, registered medical assistant certificate	1 (2)
**Occupation type** ^a^	
Medical worker (e.g., doctor, nurse, public health nurse)	26 (46)
Emergency service provider (e.g., police, firefighter, paramedic)	3 (5)
Essential service provider (e.g., work at a grocery store, gas station, postal service worker)	3 (5)
Community health worker/community health representative	14 (25)
Contact tracer or COVID-19 testing worker	7 (12)
Mental health provider (e.g., counselor, clinician, therapist, psychologist)	4 (7)
Other	13 (23)
Occupational risk of COVID-19	49 (88)
**Household Information**	
Household size	3 (0–7)
Home size (# rooms)	5 (1–18)
Children under 18 living with participant (# children)	1 (0–6)
Older adults above 65 living with participant (# adults)	0 (0–2)
Multi-generational household	6 (11)
Person in household high risk for COVID-19	29 (52)
Difficulty getting enough drinking water for household due to COVID-19	3 (5)
Able to get enough washing water for household during COVID-19	50 (89)
Home has cell service	56 (100)
Home has internet service	54 (96)
**Household finances**	
Just enough or more than enough money to make ends meet	48 (86)
Not enough or almost enough money to make ends meet	8 (14)
**Home location**	
Reservation	20 (36)
Rural (off-reservation)	16 (29)
Urban	11 (20)
Suburban	9 (16)
**Work location**	
Reservation	31 (55)
Rural (off-reservation)	6 (11)
Urban	10 (18)
Suburban	2 (4)
Other	7 (12)

^a^This was a check all that apply question, so total is more than 100%.

Participant demographics were reported in the baseline survey completed by *N* = 56 participants.

Participant demographics were only assessed at baseline, which included *N* = 56 participants. However, only 40 (71%) of the participants who completed baseline went on to complete the online training, and 38 (68%) of participants went on to complete the follow-up survey administered 1 week after completing the training. A total of *n* = 37 participants completed the final (second follow-up) survey.

### Training outcomes

Wilcoxon signed rank tests (a non-parametric comparison test) were used to examine within subject change in training outcomes over time (Table 3). Analysis revealed significant increases in PFA knowledge test scores for three out of four training modules (Module 1: Your and Your Relatives' Wellbeing; Module 3: Offering Practical Support to Community Members; and Module 4: Supporting Everyone in Our Communities) between baseline and both follow-up the one-week and three-month follow-up surveys post-PFA training completion. We did not observe significant increases in knowledge scores for PFA Module 2 (Supportive Communication in Everyday Interactions). However, baseline scores were high, indicating there was little room for a meaningful increase. At the one-week follow-up timepoint no significant change in confidence in PFA skills was observed relative to baseline. However, between baseline (*median* = 40) and the three-month follow-up survey (*median* = 41.5) there was a statistically significant increase in confidence related to PFA skills, although the change was modest (*p* = 0.046). Finally, analysis did not reveal any significant changes in measures of satisfaction with the PFA training between the two post-training follow-up timepoints; scores remained relatively stable and indicated overall high satisfaction with the training at both timepoints (28/38, 74% agreed or strongly agreed that the training was useful at the one-week follow-up and 27/33, 82% agreed or strongly agreed at the 3-month follow-up).

**Table 3:** Changes in PFA training outcomes at baseline to one-week and three-month follow-up.

**Measure**	***N***	**Baseline**	**1 Week**	**Change from baseline**	*P* value^*^
		**Median (IQR)**	**Median (IQR)**	**Median (IQR)**	
Confidence in PFA skills	32	40 (37–43)	40 (38–45)	2 (−2–4.5)	0.19
**PFA knowledge test:**					
Module 1 score	35	6 (5–7)	7 (6–8)	1 (0–2)	**<0.001**
Module 2 score	36	4 (4)	4 (4)	0 (0–0)	0.69
Module 3 score	37	3 (3, 4)	4 (4)	0 (0–1)	**<0.001**
Module 4 score	38	3 (2, 3)	3 (3)	0 (0–1)	**<0.001**
**Measure**	***N***	**Baseline**	**3 Months**	**Change from baseline**	***P*** value^*^
		**Median (IQR)**	**Median (IQR)**	**Median (IQR)**	
Confidence in PFA skills	32	40 (37–43)	41.5 (38–45)	1 (0–4.5)	0.046
**PFA knowledge test**					
Module 1 score	36	6 (5–7)	7.5 (7, 8)	1 (0–2)	**<0.001**
Module 2 score	37	4 (4)	4 (4)	0 (0–0)	>0.99
Module 3 score	36	3 (3, 4)	4 (4)	0 (0–1)	**0.01**
Module 4 score	37	3 (2, 3)	3 (3)	0 (0–1)	**<0.001**

IQR, interquartile range; PFA, Psychological First Aid.

^*^P value is from Wilcoxon signed rank test.

Bold *P* values represent results that were statistically significant (*p* < 0.05).

### Mental health outcomes

We conducted Wilcoxon signed rank tests to assess within subject change in mental health outcomes reported over time. Results showed there were no statistically significant changes in mental health outcomes between the baseline survey and one week after PFA training completion (see Table 4).

**Table 4:** Changes in mental health outcomes at baseline to one-week follow-up.

**Measure**	***N***	**Baseline**	**1 Week**	**Change from baseline**	*P* value^*^
		**Median (IQR)**	**Median (IQR)**	**Median (IQR)**	
Burnout	38	3 (2, 3)	3 (2, 3)	0 (-1- 0)	>0.99
Communal mastery	38	18.5 (15–21)	18.5 (15–21)	0 (-2-1)	0.46
Anxiety T-score	38	60.4 (56.4–63.5)	59.4 (53.2–67.7)	−1.7 (-6.1- 3.0)	0.16
MHCSF	38	45 (37–54)	48 (38–56)	2.5 (-3.0- 9.0)	0.24
Emotional wellbeing	38	11.5 (10–12)	11 (9–12)	0 (-1- 1)	0.66
Social wellbeing	38	12 (9–17)	14 (12–19)	1.5 (-2- 5)	0.07
Psychological wellbeing	38	22.5 (18–27)	23 (15–26)	0 (-3- 5)	0.51
Perceived stress	37	6 (4–8)	6 (4–8)	−1 (-2- 1)	0.40
**Brief cope**					
Problem focused coping	38	2.88 (2.50–3.14)	2.73 (2.25–3.25)	−0.06 (-0.38- 0.38)	0.57
Emotion-focused coping	38	2.42 (2.17–2.75)	2.46 (2.00–2.75)	−0.08 (-0.25- 0.25)	0.76
Avoidant coping	38	1.62 (1.38–2.00)	1.50 (1.38–2.00)	0 (-0.25- 0.12)	0.29

IQR, interquartile range.

^*^P value is from Wilcoxon signed rank test.

Table 5 reports within subject changes in mental health outcomes from baseline to the follow-up timepoint 3 months post-completion of the PFA training. Over this longer follow-up period, there was a significant increase in positive mental health from baseline (*median* = 45) to 3 months (*median* = 49), *p* = 0.03. Social wellbeing also increased significantly from baseline (*median* = 12) to 3 months (*median* = 15), *p* = 0.01. We also observed a significant decrease in burnout between baseline (*median* = 3) and three months (*median* = 2), *p* = 0.03. The analysis did not reveal significant changes in communal mastery, coping skills, anxiety, perceived stress, emotional wellbeing, or psychological wellbeing.

**Table 5:** Changes in mental health outcomes at baseline to three-months follow-up.

**Measure**	***N***	**Baseline**	**3 Months**	**Change from baseline**	*P* value^*^
		**Median (IQR)**	**Median (IQR)**	**Median (IQR)**	
Burnout	38	3 (2, 3)	2 (2, 3)	0 (−1–0)	**0.03**
Communal mastery	38	18.5 (15–21)	18.5 (16–21)	0 (−3–3)	0.72
Anxiety T-score	37	60.4 (56.4–63.5)	59.4 (56.4–65.6)	−1.0 (−5.6–3.1)	0.43
MHCSF	37	45 (37–54)	49 (44–55)	5 (−3–13)	**0.03**
Emotional wellbeing	37	11 (10–12)	12 (11–13)	1 (−1–2)	0.16
Social wellbeing	37	12 (9–16)	15 (12–18)	2 (−1–5)	**0.01**
Psychological wellbeing	37	22 (18–27)	23 (21–25)	1 (−3–6)	0.13
Perceived stress	36	6.5 (4–8.5)	6 (4–8)	−1 (−2.5–1)	0.16
**Brief cope**					
Problem focused coping	37	2.88 (2.62–3.14)	2.88 (2.50–3.25)	0.00 (−0.25–0.38)	0.91
Emotion-focused coping	37	2.42 (2.17–2.75)	2.50 (2.17–2.67)	0.00 (−0.25–0.25)	0.95
Avoidant coping	37	1.62 (1.43–2.00)	1.62 (1.38–2.00)	0 (−0.25–0.25)	0.72

IQR, interquartile range; MHCSF, Mental health continuum short form.

^*^P value is from Wilcoxon signed rank test.

Bold *P* values represent results that were statistically significant (*p* < 0.05).

### Missing data

Missing data were explored and determined to be missing not at random, with some evidence that those missing follow-up data were slightly more distressed than those that completed the study (see Supplementary Tables S1, S2). Assuming that people missing follow-up data experienced no change from their baseline levels in the sensitivity analysis (Supplementary Tables S3–S6) resulted in consistent inferences compared to the main analysis.

## Discussion

The “PFA for COVID-19 Frontline Workers in AI/AN Communities” online training was developed and evaluated with the goal of providing a culturally tailored resource to support the physical, mental, emotional, and spiritual health of frontline workers and the AI/AN communities they serve (and for many, their home community) during the pandemic. Such resources are vital given ongoing mental health inequities in many AI/AN communities (18, 35), limited and insufficient access to supportive and culturally relevant mental health care services in AI/AN communities (18, 35, 36), and the disproportionate impact of COVID-19 in AI/AN communities (5, 37). Given that the evidence base for PFA interventions is limited generally (14–17), it is noteworthy that the current study is, to our knowledge, the first pilot evaluation of PFA with AI/AN communities.

Overall, it is promising that frontline workers participating in this pilot evaluation (the majority of whom were AI/AN) reported high satisfaction with this culturally adapted PFA training. We also saw significant increases over the course of the study in PFA knowledge and confidence in using PFA skills. In a previous study of more than 26,000 state, Tribal, local, and territorial public health workers employed during the COVID-19 pandemic, approximately half reported a mental health condition (symptoms of depression, anxiety, post-traumatic stress disorder, or suicide ideation) in the past two weeks and 20% reported needing mental health services but not receiving such services (38). Therefore, widely available interventions like PFA, especially those that are culturally adapted and contextualized for a specific population, may provide short-term skills and resources for frontline workers that can support the communities they work with and themselves.

In addition, our pilot results related to mental health outcomes are promising. We observed significant increases in positive mental health and social wellbeing from baseline to 3 months among frontline workers serving AI/AN communities who completed the PFA training. While our study design did not include a control or comparison group that would allow us to attribute these findings to the PFA training specifically, pilot results showing significant change in several of our hypothesized domains is encouraging. Our culturally adapted “PFA for COVID-19 Frontline Workers in AI/AN Communities” training and guide are centered around a strengths-based theme of “caring for all our relations” (20). This focus aligns with Indigenous worldviews and teachings that mental health is integrally connected with physical, emotional, and spiritual health, as well as community and culture (39, 40). Therefore, training content reflected real AI/AN frontline worker and community experiences and design elements (e.g., photos and videos) included visuals of traditional medicines, lands, cultural traditions, and maintaining family and community connections while physical distancing. Although we cannot say with certainty, it is possible that the results we observed related to mental health outcomes were connected to completing the culturally adapted PFA training, as other research highlights the effectiveness of culturally appropriate resources in promoting AI/AN mental health and wellbeing (18, 41).

Pilot results also showed a reduction in burnout from baseline to 3 months among participating frontline workers serving AI/AN communities. Guided by focus group discussions with AI/AN COVID-19 frontline workers and our CWG, the culturally adapted PFA training/resources included an annex defining burnout and compassion fatigue and providing relaxation and self-compassion exercises (20) which could explain decreases in burnout among participants in the current study, although it is impossible to attribute these findings without a control or comparison group. We did not observe statistically significant changes in communal mastery, coping skills, perceived stress, or anxiety symptoms from the beginning of the study to either follow-up timepoints. Communal mastery scores at baseline, 1 week, and 3 months were on the higher end, indicating frontline workers already exhibited a strong sense of being able to overcome challenges because of being embedded in their community and social networks (i.e., communal mastery) (42). Previous research with AI communities has shown high rates of positive mental health while simultaneously reporting high rates of stressors (27). Our pilot results showing that frontline workers reported moderate/mild anxiety symptoms, stress, and high positive mental health mirror past research and point to the importance of attending to the full spectrum of mental health within AI/AN communities (i.e., not solely negative or positive) (27).

This study has numerous strengths. While there were helpful community and national resources offered to provide support to AI/AN communities broadly, there were few resources targeting AI/AN and other frontline workers serving AI/AN communities during COVID-19 and they were not evaluated. Importantly, the evaluated PFA resource and training was culturally adapted using community-based participatory research (CBPR) to center the voices, needs, and strengths of AI/AN communities (20). In addition, our sample included a very diverse group of frontline workers, the majority of whom were AI/AN, across age (from 20 to 79 years old), educational background (from some high school to graduate medical education), occupation (e.g., medical or mental health provider, emergency service provider, community health worker/community health representative), and home/work location (e.g., reservation, rural/off-reservation, urban). It is promising that this diverse group of frontline workers reported high satisfaction with this culturally adapted PFA intervention, demonstrating the importance of culturally tailored resources for promoting mental health.

While this resource is novel, this study also has important limitations. We had a small sample size, with mostly female participants, and some attrition between baseline and follow-up timepoints, limiting our interpretation and the generalizability of the current findings broadly to AI/AN communities or other U.S. groups. There are 574 federally recognized Tribes, 63 state recognized Tribes, and urban AI/AN communities across the U.S. all with unique community and cultural contexts, as well as different experiences during the COVID-19 pandemic. Therefore, our study is not representative of frontline workers across all Tribal and urban AI/AN communities. In addition, our culturally adapted PFA intervention was developed as an online resource and training. Given that 18% of those residing on Tribal reservations have no home internet access (43), it is possible that access to internet posed a recruitment barrier to other potential participants in AI/AN communities accessing the online PFA training and resources for frontline workers. While online delivery of the PFA training was acceptable to participants in this study via satisfaction ratings, we acknowledge this format may leave out important groups of frontline workers who may need it most. Because the training and study were delivered entirely online, we were unable to measure trainees' ability to implement PFA skills in practice. With guidance and input from the CWG, we attempted to assess this proximately by asking participants to self-report confidence in applying PFA skills, and results showed important increases in this confidence following completion of the online PFA training.

Moreover, in the absence of a control or comparison condition, it is impossible to attribute significant changes we observed in this pilot evaluation to participating in the PFA training, as these changes may simply represent regression toward the mean, rather than change due to the intervention. While we did observe statistically significant changes in certain positive mental health and social outcomes over the follow-up period, other outcomes did not change (i.e., anxiety, coping skills, communal mastery, perceived stress). In addition, we did not see significant changes in any study variables from baseline to 1 week post-PFA training. The world was in a state of continual change over time from a global health emergency to the end of COVID-19 as a global pandemic (44). Therefore, it is possible that some changes observed (e.g., burnout) could have been related to other variables, such as time passing, rather than the PFA training. A follow-up period longer than 3 months would have also allowed for more information on whether the observed positive changes persisted over time. Future research should employ an experimental design or other type of design to enhance internal validity (e.g., randomized controlled trial) with a larger sample size to determine whether this culturally adapted PFA training has an impact on frontline workers' mental health.

We intend to keep the “PFA for COVID-19 Frontline Workers in AI/AN Communities” resource guide and online training available to the public through the CIH website (45). While the U.S. and world are no longer facing a global pandemic, aspects of this training will continue to be relevant for those serving AI/AN communities. For example, identifying signs of stress, learning ways to cope with stress, and offering mental health resources and practical support to community members will continue to serve as important tools for a variety of professionals serving AI/AN communities (20). Aligning with CBPR approaches, we plan to disseminate pilot results through CIH social media channels and website to ensure these results and the PFA guide and training are shared with frontline workers and AI/AN communities. When developing the PFA resource guide and training and planning our pilot evaluation study, our CWG who guided this project shared the importance of centering Inter-Tribal teachings and cultural values of caring for all of our relatives. We continue to be inspired and grateful to the COVID-19 frontline workers, as well as every person who continues to serve AI/AN communities today to promote the health, mental health, and wellbeing of all of our relatives. Future research should continue to pursue adaptation and creation of culturally tailored and grounded resources to support mental health and wellbeing among AI/AN frontline workers and communities.

## Data availability statement

The datasets presented in this article are not readily available because permission must be given by the Navajo Nation Human Research Review Board. Requests to access the datasets should be directed to the corresponding author(s).

## Ethics statement

The studies involving humans were approved by Johns Hopkins Bloomberg School of Public Health Institutional Review Board and the Navajo Nation Human Research Review Board. The studies were conducted in accordance with the local legislation and institutional requirements. Written informed consent for participation was not required from the participants or the participants' legal guardians/next of kin because informed consent was obtained from participants via an online survey.

## Author contributions

VO'K: Conceptualization, Investigation, Supervision, Writing – original draft, Writing – review & editing, Funding acquisition. FG: Conceptualization, Data curation, Formal analysis, Investigation, Project administration, Supervision, Visualization, Writing – original draft, Writing – review & editing. NV: Writing – original draft, Writing – review & editing. TM: Investigation, Writing – review & editing. MC: Investigation, Writing – review & editing. SN: Conceptualization, Writing – review & editing. SJ: Conceptualization, Writing – review & editing. MAC: Conceptualization, Writing – review & editing. KC: Data curation, Formal analysis, Validation, Writing – review & editing. AB: Funding acquisition, Writing – review & editing. EH: Conceptualization, Investigation, Validation, Writing – review & editing.
