# Supplementary material for: Pilot evaluation of a Psychological First Aid online training for COVID-19 frontline workers in American Indian/Alaska Native communities
Source: Front Public Health. 2024 Jun 27;12:1346682. doi: 10.3389/fpubh.2024.1346682 (PMC11240286; doi:10.3389/fpubh.2024.1346682)
Supplement: Supplementary file 1 [file Table_1.DOCX]

| Table S1. Characteristics of participants with and without 3-month follow-up | | | |
| --- | --- | --- | --- |
| Results presented are N (%) or median (interquartile range). | | | |
| Characteristic | With 3-month follow-up  N=38 | Missed 3 month follow-up  N=18 | P value* |
| Gender: |  |  | 0.76 |
| Female | 32 (84) | 17 (94) |  |
| Male | 5 (13) | 1 (6) |  |
| Other | 1 (3) | 0 (0) |  |
| Age category: |  |  | 0.56 |
| 20-29 years | 3 (8) | 3 (17) |  |
| 30-39 years | 15 (39) | 6 (33) |  |
| 40-49 years | 11 (29) | 3 (17) |  |
| 50-59 years | 7 (18) | 4 (22) |  |
| 60-69 years | 2 (5) | 1 (6) |  |
| 70-79 years | 0 (0) | 1 (6) |  |
| Marital status: |  |  | 0.48 |
| Single | 13 (34) | 7 (39) |  |
| Living with partner | 3 (8) | 4 (22) |  |
| Legally married | 17 (45) | 6 (33) |  |
| Divorced | 3 (8) | 0 (0) |  |
| Widowed | 2 (5) | 1 (6) |  |
| Education: |  |  | 0.12 |
| Some high school | 0 (0) | 1 (6) |  |
| High school diploma | 2 (5) | 0 (0) |  |
| Some college | 2 (5) | 4 (22) |  |
| Associate’s degree | 6 (16) | 5 (22) |  |
| Bachelor’s degree | 15 (39) | 4 (22) |  |
| Graduate education | 7 (18) | 4 (22) |  |
| Graduate medical education | 5 (13) | 0 (0) |  |
| Other, registered medical assistant certificate | 1 (3) | 0 (0) |  |
| Occupation type^a^: |  |  |  |
| Medical worker (e.g., doctor, nurse, public health nurse) | 21 (55) | 5 (28) | 0.08 |
| Emergency service provider (e.g., police, firefighter, paramedic) | 1 (3) | 2 (11) | 0.24 |
| Essential service provider (e.g., work at a grocery store, gas station, postal service worker) | 2 (5) | 1 (6) | >0.99 |
| Community health worker/Community health representative | 5 (13) | 9 (50) | **0.006** |
| Contact tracer or COVID-19 testing worker | 5 (13) | 2 (11) | >0.99 |
| Mental health provider (e.g., counselor, clinician, therapist, psychologist) | 3 (8) | 1 (6) | >0.99 |
| Other | 9 (24) | 4 (22) | >0.99 |
| Occupational risk of COVID-19 | 32 (84) | 17 (94) | 0.68 |
| Household Information |  |  |  |
| Household size | 3 (1-3) | 3 (2-5) | 0.22 |
| Home size (# rooms) | 5 (3-8) | 4.5 (3-6) | 0.39 |
| Children under 18 living with participant (# children) | 1 (0-2) | 1 (0-2) | 0.70 |
| Older adults above 65 living with participant (# adults) | 0 (0-0) | 0 (0-0) | 0.06 |
| Multi-generational household | 1 (3) | 5 (28) | **0.01** |
| Person in household high risk for COVID-19 | 17 (45) | 12 (67) | 0.25 |
| Difficulty getting enough drinking water for household due to COVID-19 | 2 (5) | 1 (6) | 0.49 |
| Able to get enough washing water for household during COVID-19 | 35 (92) | 15 (83) | 0.37 |
| Home has cell service | 38 (100) | 18 (100) | - |
| Home has internet service | 38 (100) | 16 (89) | 0.10 |
| Household finances: |  |  | 0.70 |
| Just enough or more than enough money to make ends meet | 33 (87) | 15 (83) |  |
| Not enough or almost enough money to make ends meet | 5 (13) | 3 (17) |  |
| Home location: |  |  | 0.24 |
| Reservation | 10 (26) | 10 (56) |  |
| Rural (off-reservation) | 12 (32) | 4 (22) |  |
| Urban | 9 (24) | 2 (11) |  |
| Suburban | 7 (18) | 2 (11) |  |
| Work location: |  |  | 0.29 |
| Reservation | 22 (58) | 9 (50) |  |
| Rural (off-reservation) | 4 (11) | 2 (11) |  |
| Urban | 6 (16) | 4 (22) |  |
| Suburban | 0 (0) | 2 (11) |  |
| Other | 6 (16) | 1 (6) |  |
| ^a^ This was a check all that apply question, so total is more than 100% | | | |

| Table S2. Comparison of baseline measures for participants with and without 3-month follow-up | | | |
| --- | --- | --- | --- |
| Results presented are median (interquartile range). | | | |
| Measure | **With 3-month**  **follow-up** | **Missed 3-month**  **follow-up** | **P value*** |
| Number of participants, N (%) | 38 (68) | 18 (32) |  |
| Burnout | 3 (2-3) | 3 (2-4) | 0.38 |
| Communal mastery score | 18.5 (15-21) | 21.5 (18-24) | **0.01** |
| Brief cope: |  |  |  |
| Problem focused coping | 2.88 (2.50-3.14) | 2.65 (2.25-3.12) | 0.17 |
| Emotion focused coping | 2.42 (2.17-2.75) | 2.47 (2.25-2.83) | 0.59 |
| Avoidant coping | 1.62 (1.38-2.00) | 1.94 (1.5-2.5) | 0.09 |
| Anxiety T-score | 60.4 (56.4-63.5) | 62.5 (56.4-70.8) | 0.14 |
| MHCSF Total | 45 (37-54) | 43.5 (35-52) | 0.54 |
| MHCSF Flourish | 1 (0-1) | 0 (0-1) | 0.18 |
| MHCSF Languish | 0 (0-0) | 0 (0-0) | 0.98 |
| MHCSF Emotional | 11.5 (10-12) | 10 (7-12) | 0.19 |
| MHCSF Social | 12 (9-17) | 13 (7-15) | 0.74 |
| MHCSF Psychological | 22.5 (18-27) | 21.5 (17-25) | 0.76 |
| Perceived stress score | 6 (4-8) | 7.5 (3-10) | 0.47 |
| Confidence in PFA skills | 40 (37-43) | 41.5 (39-45) | 0.25 |
| PFA Knowledge test: |  |  |  |
| Module 1 | 6 (5-7) | 6 (5-7) | 0.96 |
| Module 2 | 4 (4-4) | 4 (3-4) | 0.08 |
| Module 3 | 3 (3-4) | 3 (3-4) | 0.98 |
| Module 4 | 3 (2-3) | 3 (2-3) | 0.62 |
| * P values are from Wilcoxon rank sum test | | | |

| Table S3. Sensitivity analysis* comparing change from baseline in mental health outcomes at 1 week | | | | |
| --- | --- | --- | --- | --- |
| Measure | **Change from baseline** | **P value**** | **Change from baseline*** | **P value**** |
|  | Median (IQR)  N=38 |  | Median (IQR)  N=56 |  |
| Burnout | 0 (-1- 0) | >0.99 | 0 (0- 0) | >0.99 |
| Communal mastery | 0 (-2- 1) | 0.46 | 0 (-1.5- 1) | 0.46 |
| Brief cope: |  |  |  |  |
| Problem focused coping | -0.06 (-0.38- 0.38) | 0.57 | 0.0 (-0.25- 0.19) | 0.57 |
| Emotion-focused coping | -0.08 (-0.25- 0.25) | 0.76 | 0.0 (-0.17- 0.14) | 0.76 |
| Avoidant coping | 0 (-0.25- 0.12) | 0.29 | 0 (-0.12- 0.00) | 0.29 |
| Anxiety T-score | -1.7 (-6.1- 3.0) | 0.16 | 0 (-2.8- 0.0) | 0.16 |
| MHCSF | 2.5 (-3- 9) | 0.24 | 0 (-1.5- 4) | 0.24 |
| Emotional well-being | 0 (-1- 1) | 0.66 | 0 (-1- 1) | 0.66 |
| Social well-being | 1.5 (-2- 5) | 0.07 | 0 (0- 3) | 0.07 |
| Psychological well-being | 0 (-3- 5) | 0.51 | 0 (-1- 2) | 0.51 |
| Perceived stress | -1 (-2- 1) | 0.40 | 0 (-1- 0) | 0.40 |
| * Substituting 0 (no change) for those missing data at 1 week  ** P value is from Wilcoxon signed rank test | | | | |

| Table S4. Sensitivity analysis* comparing change from baseline in mental health outcomes at 3 months | | | | |
| --- | --- | --- | --- | --- |
| Measure | **Change from baseline** | **P value**** | **Change from baseline*** | **P value**** |
|  | Median (IQR)  N=36-38 |  | Median (IQR)  N=56 |  |
| Burnout | 0 (-1- 0) | 0.03 | 0 (0- 0) | 0.03 |
| Communal mastery | 0 (-3- 3) | 0.72 | 0 (-1- 1.5) | 0.72 |
| Brief cope: |  |  |  |  |
| Problem focused coping | 0.00 (-0.25- 0.38) | 0.91 | 0 (-0.12- 0.12) | 0.91 |
| Emotion-focused coping | 0.00 (-0.25- 0.25) | 0.95 | 0 (-0.08- 0.12) | 0.95 |
| Avoidant coping | 0 (-0.25- 0.25) | 0.72 | 0 (-0.12- 0.12) | 0.72 |
| Anxiety T-score | -1.0 (-5.6- 3.1) | 0.43 | 0 (-3.0- 3.0) | 0.43 |
| MHCSF | 5 (-3- 13) | 0.03 | 0 (0- 9.5) | 0.03 |
| Emotional well-being | 1 (-1- 2) | 0.16 | 0 (0- 1) | 0.16 |
| Social well-being | 2 (-1- 5) | 0.01 | 0 (0- 4) | 0.01 |
| Psychological well-being | 1 (-3- 6) | 0.13 | 0 (0- 2.5) | 0.13 |
| Perceived stress | -1 (-2.5- 1) | 0.16 | 0 (-1.5- 0) | 0.16 |
| * Substituting 0 (no change) for those missing data at 3 months  ** P value is from Wilcoxon signed rank test | | | | |

| Table S5. Sensitivity analysis* comparing change from baseline in training outcomes at 1 week | | | | |
| --- | --- | --- | --- | --- |
| Measure | **Change from baseline** | **P value**** | **Change from baseline*** | **P value**** |
|  | Median (IQR)  N=32-38 |  | Median (IQR)  N=56 |  |
| Confidence in PFA skills | 2 (-2- 4.5) | 0.19 | 0 (0- 2) | 0.19 |
| PFA knowledge test: |  |  |  |  |
| Module 1 score | 1 (0- 2) | <0.001 | 0 (0- 1) | <0.001 |
| Module 2 score | 0 (0- 0) | 0.69 | 0 (0- 0) | 0.69 |
| Module 3 score | 0 (0- 1) | <0.001 | 0 (0- 1) | <0.001 |
| Module 4 score | 0 (0- 1) | <0.001 | 0 (0- 0.5) | <0.001 |
| * Substituting 0 (no change) for those missing data at 1 week  ** P value is from Wilcoxon signed rank test | | | | |

| Table S6. Sensitivity analysis* comparing change from baseline in training outcomes at 3 months | | | | |
| --- | --- | --- | --- | --- |
| Measure | **Change from baseline** | **P value**** | **Change from baseline*** | **P value**** |
|  | Median (IQR)  N=32-37 |  | Median (IQR)  N=56 |  |
| Confidence in PFA skills | 1 (0 – 4.5) | 0.046 | 0 (0- 1.5) | 0.046 |
| PFA knowledge test: |  |  |  |  |
| Module 1 score | 1 (0- 2) | <0.001 | 0 (0- 2) | <0.001 |
| Module 2 score | 0 (0- 0) | >0.99 | 0 (0- 0) | >0.99 |
| Module 3 score | 0 (0- 1) | 0.01 | 0 (0- 1) | 0.01 |
| Module 4 score | 0 (0- 1) | <0.001 | 0 (0- 0) | <0.001 |
| * Substituting 0 (no change) for those missing data at 3 months  ** P value is from Wilcoxon signed rank test | | | | |
